# Surgeon-performed transcutaneous laryngeal ultrasound for vocal cord assessment after total thyroidectomy: a prospective study: Original article

**DOI:** 10.1007/s00423-024-03362-4

**Published:** 2024-06-11

**Authors:** Leonardo Rossi, Piermarco Papini, Andrea De Palma, Lorenzo Fregoli, Chiara Becucci, Carlo Enrico Ambrosini, Riccardo Morganti, Gabriele Materazzi

**Affiliations:** 1 Department of Surgical, Medical and Molecular Pathology and Critical Area, University of Pisa, Pisa, Italy; 2 Section of Statistics, University of Pisa, Pisa, Italy

**Keywords:** Thyroidectomy, Transcutaneous laryngeal ultrasound, Recurrent laryngeal nerve Injury, Vocal cord Palsy, Vocal Cord movement impairment, Ultrasonography, Flexible nasolaryngoscopy

## Abstract

**Purpose:**

Assessing vocal cord mobility is crucial for patients undergoing thyroid surgery. We aimed to evaluate the feasibility and efficacy of surgeon-performed transcutaneous laryngeal ultrasound (TLUS) compared to flexible nasolaryngoscopy.

**Method:**

From February 2022 to December 2022, we conducted a prospective observational study on patients scheduled for total thyroidectomy at our Institution. All patients underwent TLUS followed by flexible nasolaryngoscopy by a blinded otolaryngologist. Findings were classified as normal or vocal cord movement impairment and then compared. Patients evaluable on TLUS were included in Group A, while those not evaluable were included in Group B, and their features were compared.

**Results:**

Group A included 180 patients, while Group B included 21 patients. Male sex (*p* < 0.001), age (*p* = 0.034), BMI (*p* < 0.001), thyroid volume (*p* = 0.038), and neck circumference (*p* < 0.001) were associated with Group B. TLUS showed a sensitivity, specificity, positive predictive value, negative predictive value, and accuracy of 100%, 99.4%, 94.4%, 100%, and 99.4%, respectively. Cohen’s K value was 0.984.

**Conclusion:**

TLUS is a valid, easy-to-perform, non-invasive, and painless alternative for evaluating vocal cords in selected patients. It can be used either as a first level exam and as screening tool for selecting cases for flexible nasolaryngoscopy. TLUS should be integrated into routine thyroid ultrasound examination.

## Introduction

Recurrent laryngeal nerve injury is one of the most worrisome complications following thyroid surgery, with transient forms ranging from 1 to 30% and permanent forms from 0.5 to 5% [1]. Additionally, it often leads to medicolegal issues [2]. This injury is associated with vocal fold movement impairment (VFMI), which can result in dysphonia. Bilateral occurrences can lead to life-threatening airway obstruction, potentially necessitating tracheostomy. However, VFMI may also manifest asymptomatically [3].

Flexible nasolaryngoscopy (FNL) is the gold standard technique for assessing vocal cord (VC) mobility [4], providing visualization in over 99% of cases [5]. Nevertheless, FNL is an invasive procedure associated with discomfort or pain [6], often requiring referral to an otolaryngologist specialist, incurring both time and financial expenses. Transcutaneous laryngeal ultrasound (TLUS) has been proposed as an alternative method for assessing VC mobility. However, this technique remains underutilized, with few studies reporting contrasting findings with variable sensitivity and specificity [3, 6–14].

The aim of this study was to assess the feasibility and effectiveness of surgeon-performed TLUS for evaluating VC mobility compared to FNL in patients who underwent total thyroidectomy.

## Materials and methods

Between February 2022 and December 2022, we conducted a prospective observational transversal study at our Institution. Participants were selected by opening a sealed envelope from among patients who had undergone total thyroidectomy with or without lymph node dissection. Exclusion criteria included any less than total thyroidectomy (e.g., lobectomy or isthmusectomy), age under 18 years old, history of vocal cord disease or surgery, and presence of tracheostomy. All enrolled patients underwent TLUS on post-operative day 1 or 2, followed immediately by evaluation via laryngoscopy by a blinded otolaryngologist for those patients who were evaluable on TLUS. Patients remained silent during the exams to prevent bias in voice quality assessment. Ultrasonographic and laryngoscopic findings were categorized as normal or VFMI and were then compared and classified as concordant or discordant. Additionally, patients were divided into two groups based on VC visualization during TLUS: Group A included patients with evaluable vocal cords, while Group B included those with non-evaluable vocal cords. The features of the two groups were compared.

All TLUS were conducted by an experienced investigator skilled in neck ultrasound (LR) to minimize variability in VC assessment. FNL procedures were performed by experienced examiners who defined VFMI as any deviation from normal VC mobility, including VC weakness with reduced movements, VC asymmetry, and complete immobility.

Data were collected in a prospectively maintained electronic database, including patients’ age, sex, body mass index (BMI), neck length, neck circumference, ultrasound-estimated thyroid volume (UETV), presence of thyroid cartilage calcification, ability to visualize vocal cords, normal VC mobility or VFMI on TLUS, and post-operative FNL findings.

The sensitivity, specificity, positive predictive value (PPV), negative predictive value (NPV), and accuracy of TLUS in identifying VFMI were determined. True positive was defined when VFMI was documented on either TLUS or FNL; true negative was defined when both TLUS and FNL documented normal VC mobility; false positive was defined when TLUS showed VFMI but FNL documented normal VC mobility; false negative was defined when TLUS showed normal VC mobility but FNL documented VFMI.

The primary objective of the study was to evaluate the efficacy of surgeon-performed TLUS for assessing VC mobility compared to FNL in patients underwent total thyroidectomy. The secondary objective was to identify factors influencing the evaluability of vocal cords on TLUS.

This study was conducted in accordance with the Declaration of Helsinki and was approved by the Institutional Review Board (IRB) of Area Vasta Nord-Ovest (IRB code: 23915_Materazzi).

### Transcutaneous Laryngeal Ultrasonography

TLUS was conducted using a 4–13-MHz linear probe (Esaote MyLab Twice; Esaote S.p.A, Genova, Italy). Patients lay supine with their necks slightly extended during the assessment. An ultrasound transducer was positioned transversely over the anterior aspect of the middle portion of the thyroid cartilage, serving as acoustic window. The transducer scanned caudo-cranially until both vocal cords were assessable. False cords and arytenoids were identified whenever possible, serving as sonographic landmarks.

**Fig. 1:**
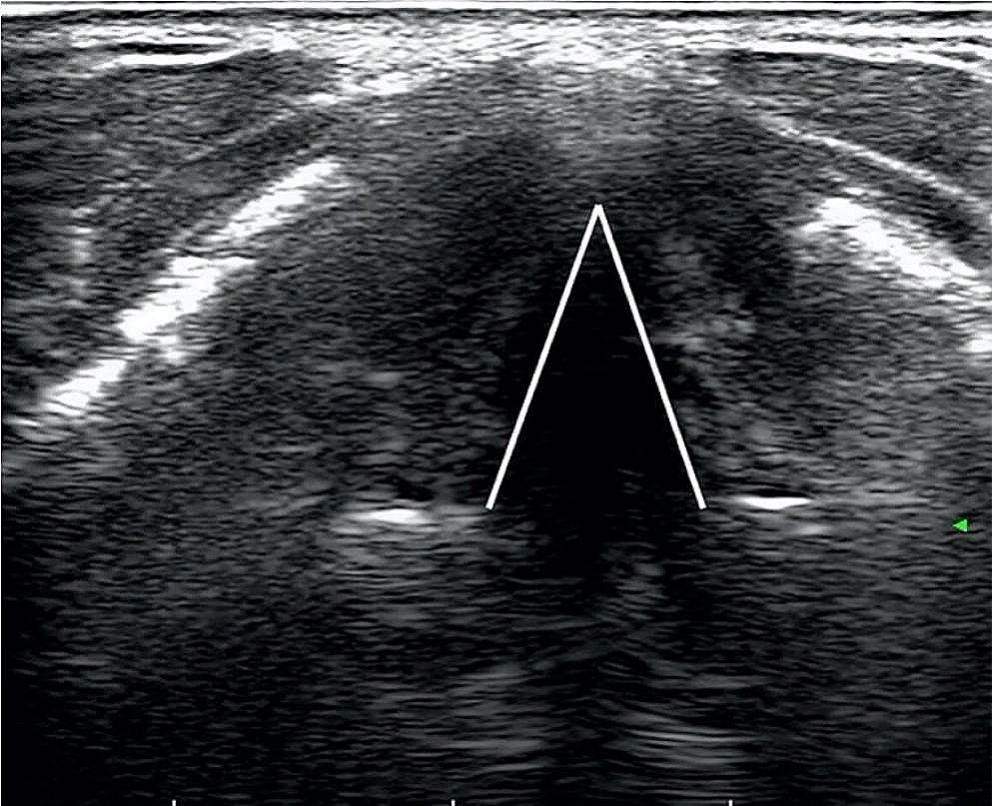
TLUS shows bilateral normal vocal cords abduction

To optimize the image, the grayscale was adjusted until false cords became hyperechoic, while true cords became hypoechoic. VC mobility was evaluated during passive spontaneous breathing, active phonation with a sustained vowel ‘‘ee,” and Valsalva maneuver. Normal mobility of the true vocal cords was characterized by symmetrical movement in adduction and abduction (Fig. 1).

On the contrary, according to the definition used for FNL, we interpreted VFMI as any deviation from normal mobility, including VC weakness with reduced movements, asymmetry, and complete immobility.

**Fig. 2:**
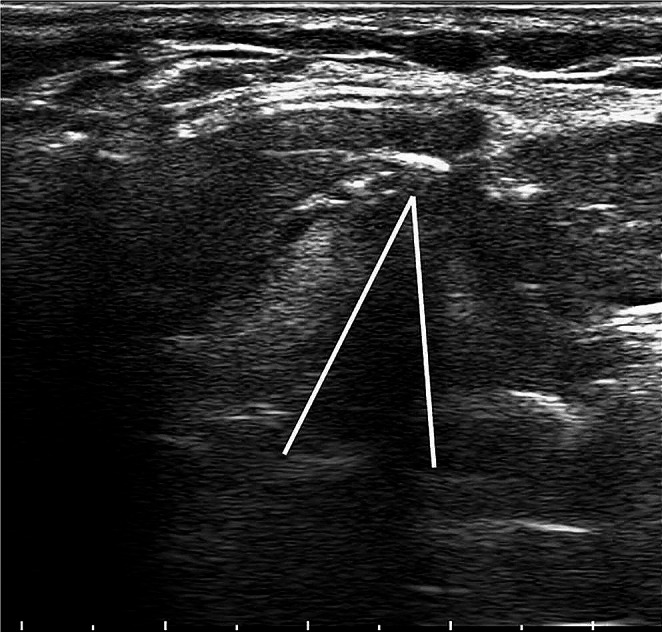
TLUS shows left VFMI

During the Valsalva maneuver, in the case of normal mobility, vocal cords appeared symmetrically adducted in the midline. Conversely, when patients relaxed from the Valsalva maneuver, vocal cords would abduct in the case of normal mobility, whereas they appeared still or with decreased/passive movement in the case of VFMI (Fig. 2).

### Statistical analysis

Categorical data were described using absolute and relative frequencies (%), while continuous data were summarized with the mean value and standard deviation.

Statistical analysis utilized the Chi-square test for categorical variables and the independent samples t-test (two-tailed) for continuous variables. Sensitivity, specificity, PPV, NPV, and accuracy were assessed based on FNL findings. Concordance between TLUS and FNL was evaluated using Cohen’s K value.

Significance was set at 0.05 with a 95% confidence interval (CI). All analyses were conducted using Microsoft Excel and SPSS v.28 software (IBM Corp., Armonk, NY, USA).

The target sample size was 180 patients, providing 80% power at the 5% (two-sided) level of significance, with an effect size of 3% in detecting VFMI (TLUS vs. FNL) associated with a Cohen’s K value of 0.800.

## Results

The study included 201 patients, of whom 167 (83.1%) were females and 34 (16.9%) were males. Overall, TLUS enabled visualization of vocal cords in 180 patients (89.6%) (Group A patients). Male gender was found to be negatively associated with vocal cord evaluability on TLUS: all females were assessable, whereas only 13 (38.2%) males were assessable (*p* < 0.001). All Group B patients (*n* = 21) presented thyroid cartilage calcification, which was always absent in Group A (*p* < 0.001). Additionally, a statistically significant difference was observed between Group A and Group B in terms of age (49 ± 13 and 56 ± 9 years, respectively; *p* = 0.034), BMI (25.4 ± 5.5 and 29.8 ± 5.5 kg/m², respectively; *p* < 0.001), UETV (38.9 ± 33.6 and 60.1 ± 57.7 ml, respectively; *p* = 0.038), and neck circumference (36.5 ± 3.8 and 43.0 ± 3.6 cm, respectively; *p* < 0.001). No statistically significant difference was found in terms of neck length between Group A and Group B (14.1 ± 2.4 and 14.3 ± 2.1 cm, respectively; *p* = 0.693). These findings are summarized in Table 1.

**Table 1:** Patients’ features

Variables	Overall (*n* = 201)	Group A (*n* = 180)	Group B (*n* = 21)	*P*-value
**Female**, n (%)	167	167 (100.0%)	0 (0.0%)	< 0.001
**Male**, n (%)	34	13 (38.2%)	21 (61.8%)	
**Age**, mean ± sd; years	50 *±* 13	49 *±* 13	56 *±* 9	0.034
**BMI**, mean ± sd; kg/m²	25.9 ± 5.6	25.4 *±* 5.5	29.8 *±* 5.5	< 0.001
**UETV**, mean ± sd; ml	40.3 ± 35.9	38.9 ± 33.6	60.1 *±* 57.7	0.038
**Neck Length**, mean ± sd; cm	14.1 ± 2.4	14.1 *±* 2.4	14.3 *±* 2.1	0.693
**Neck Circumference**, mean ± sd; cm	37.2 ± 4.2	36.5 *±* 3.8	43.0 *±* 3.6	< 0.001

BMI: body mass index; UETV: ultrasound-estimated thyroid volume; sd: standard deviation

TLUS identified postoperative unilateral VFMI in 18 patients (10%); however, FNL confirmed VFMI in only 17 patients (9.4%), with one case showing normal VC mobility (false positive) (Table 2). Notably, only 12 patients (6.7%) were dysphonic, indicating that 29.4% of cases were underdiagnosed clinically.

**Table 2:** TLUS and FNL findings of patients with evaluable vocal cords

	TLUS		
**FNL**	Normal, n	VFMI, n	Total, n (%)
Normal, n	162	1	163 (90.6%)
VFMI, n	0	17	17 (9.4%)
Total, n (%)	162 (90%)	18 (10%)	180

FNL: flexible nasolaryngoscopy; TLUS: transcutaneous laryngeal ultrasound; VFMI: vocal Fold movement impairment

The sensitivity of TLUS was found to be 100%, with a specificity of 99.4%, a PPV of 94.4%, a NPV of 100%, and an accuracy of 99.4%. Additionally, the concordance between TLUS and FNL was excellent, with a Cohen’s K value of 0.984 (Table 3).

**Table 3:** Concordance between TLUS and FNL.

Parameter	Value (95% CI)
**Sensitivity**, %	100 (80.5–100)
**Specificity**, %	99.4 (96.6–100)
**PPV**, %	94.4 (70.7–99.2)
**NPV**, %	100
**Accuracy**, %	99.4 (96.9–100)
**Cohen’s K value** (0–1)	0.984

PPV: positive predictive value; NPV: negative predictive value

Nonetheless, in one patient, TLUS reported a left VFMI, while FNL diagnosed a right VFMI. Although both techniques documented “unilateral VFMI,” we may consider this case as a double error (both false positive and false negative). However, even when considering the right and left vocal cords separately, TLUS demonstrated an accuracy of 98.9% and 99.4%, with Cohen’s K values of 0.944 and 0.975, respectively.

## Discussion

Since its introduction, TLUS has been proposed as an alternative method for assessing VC mobility in patients scheduled for thyroid surgery [15]. Despite some encouraging studies [3, 8, 15, 16], its use in clinical practice is currently limited.

The American Academy of Otolaryngology and Head and Neck Surgery Guidelines recommend that all patients undergoing thyroid and parathyroid surgery should undergo preoperative and postoperative FNL [4]. However, many surgeons consider routine FNL unnecessary and reserve it for selected cases [9, 17].

Furthermore, the American Thyroid Association Management Guidelines suggest that patients should undergo a postoperative laryngeal examination in case of voice abnormality [18]. However, voice quality change is not reliable indicator of VFMI, and a normal voice does not exclude the possibility of recurrent laryngeal nerve injury [15, 19]. Accordingly, in our case series, only 12 out of 17 (70.6%) patients with VFMI presented with dysphonia. This finding theoretically leads to underestimate about 30% of overall recurrent laryngeal nerve injury.

In our experience, TLUS has proven to be an effective and reliable technique for visualizing vocal cords, with 89.6% of patients being evaluable with vocal cords clearly identified. Moreover, TLUS appears to be particularly effective in female patients, who represent the majority of candidates for thyroidectomy, with a 100% evaluability rate in our study. However, this method only enables VC visualization in 38.2% of male patients, making it inadequate as an alternative to FNL in this cohort (*p* < 0.001). This discrepancy is attributed to thyroid cartilage calcification and its acute angle, which are features mainly encountered in male patients. Consistent with our findings, previous studies have also described differences in evaluability rates between male and female patients [3, 8].

Nevertheless, some authors have proposed a novel lateral approach to overcome the limited efficacy of TLUS in male patients [3, 20]. Woo et al. reported a 100% visualization rate in male patients using this alternative technique [20]. Additionally, the use of a gel pad has been introduced to improve surface contact between the ultrasound probe and the thyroid cartilage [21, 22]. Knyazeva et al. reported that the use of a gel pad increased visualization rates in male patients from 35 to 78% and recommended its routine use [22]. Unfortunately, the lateral approach and the gel pad were not tested in our study but will be evaluated in future studies.

Male gender was not the only factor negatively influencing VC evaluability during TLUS. We found that age (*p* = 0.034), BMI (*p* < 0.001), UETV (*p* = 0.038), and neck circumference (*p* < 0.001) were also statistically associated with patients being deemed not evaluable. Similarly, Wong et al. [23] reported that, in multivariate analysis, age, male sex, body height, and the distance from the collar incision to the thyroid cartilage were independent factors for unevaluable vocal cords.

In our study, when vocal cords were assessable, TLUS proved to be an effective and reliable technique for evaluating VC mobility. We documented only 1 false positive, and no VFMI were missed on ultrasound, resulting in a sensitivity of 100%, specificity of 99.4%, PPV of 94.4%, NPV of 100%, and accuracy of 99.4%. Moreover, the concordance between TLUS and FNL was excellent, with a Cohen’s K value of 0.984.

Our optimal results align with previous studies [3, 7–12]. In particular, Wong et al. conducted a large series involving 1000 patients and documented a visualization rate of 92.4% with a sensitivity of 87.5% [7]. The authors concluded that TLUS could prevent 87.7% of high-risk patients from requiring laryngoscopy and recommended incorporating this technique into the ultrasound examination of the thyroid. Similarly, Gambardella et al. [3], in a prospective study involving 396 patients evaluated by TLUS, reported a sensitivity of 96.8%, specificity of 95.6%, PPV of 65.2%, and NPV of 99.7%. However, these two large studies only assessed VC mobility before thyroidectomy when VC palsy is extremely rare, limiting statistical power.

Additionally, Knyazeva et al. in 2018 conducted a large retrospective study involving 668 patients enrolled in two tertiary referral centers of endocrine surgery, examining the performance of TLUS in thyroidectomized patients. The authors reported excellent results, with a postoperative TLUS sensitivity of 86%, specificity of 99.1%, PPV of 89.4%, and NPV of 98.7% [8].

On the contrary, some studies on TLUS reported inferior results [6, 13, 14]. Sidhu et al. analyzed 107 patients using TLUS, which identified 8 out of 13 vocal cord palsies. Overall, TLUS demonstrated a sensitivity of 62%, specificity of 97%, PPV of 73%, and NPV of 95% for detecting VC paralysis. The authors concluded that TLUS is not a reliable alternative to laryngoscopy [13]. Similarly, Kandil et al., in a study involving 250 patients, claimed that TLUS should not be considered as an alternative to FNL, especially in overweight and obese patients and in the postoperative setting. The authors documented only about 50% of VC palsy using TLUS, with a sensitivity, specificity, and accuracy of 55.6%, 38.7%, and 39.6%, respectively. However, the authors defined false positive results as the inability to detect mobile vocal cords and false negative results as the inability to detect a confirmed VC palsy. This led to a high rate of false positive and false negative results associated with the low evaluability rate [14].

Moreover, Borel et al. reported unsatisfactory results of TLUS with a sensitivity of 33% and an NPV of 95% for identifying VC palsy in patients undergoing thyroidectomy [6]. However, in this study, the sonographic examination was performed by radiologists who had no prior experience with TLUS.

Recently, Kim et al. published a meta-analysis to evaluate the efficacy and reliability of TLUS in assessing VC function [24]. The authors included 17 studies in their analysis, most of which reported promising results. Overall, the VC visualization rate was excellent (95.7%), as were the sensitivity (91.5%), specificity (97.7%), and NPV (99.2%). Despite some heterogeneities in methods among the scrutinized papers, the authors concluded that TLUS is diagnostically accurate regardless of the landmarks used, VC palsy definition, and timing for applying ultrasonography [24].

In our experience, TLUS is an effective, reliable, quick (lasting only 1–2 min), and non-invasive alternative to FNL. It can be performed during both pre-operative and post-operative ultrasonography without additional costs. Moreover, it may be cost-effective, reducing the need for unnecessary FNL in many patients, including those who are apprehensive about this approach, when the VCs are adequately visualized and fully mobile on TLUS. However, in cases of difficult VC visualization, abnormal mobility on ultrasound, loss of signal during intraoperative nerve monitoring, dysphonia, or redo surgery, we believe that FNL is still mandatory.

Our study harbors limitations. Firstly, it lacks quantitative measurements, such as vocal fold displacement velocity (VFDV) with pulsed Doppler, which could have provided additional objectivity to the findings. VFDV is proportional to the velocity of the wave causing vibrations of the vocal folds, and after VFMI, it should be significantly reduced [25]. Additionally, we did not test the application of a gel pad, and TLUS was not performed using a lateral approach. These techniques will be evaluated in future studies, with the hope of obtaining better results, especially in adult male patients. Lastly, this study represents the experience of a single surgeon, and therefore, these findings may not be easily generalizable to other practitioners.

## Conclusion

In conclusion, TLUS emerges as a cost-effective, rapid, non-invasive, and effective method for assessing VC mobility in patients undergoing total thyroidectomy. Its utility is particularly notable in young women, who constitute the majority of candidates for thyroid surgery, and it could serve as a screening tool, reserving FNL for unevaluable cases or those with abnormal VC mobility. Incorporating TLUS into routine thyroid ultrasound examinations could enhance the efficiency and accuracy of utrasound assessment in these patients.

## Data Availability

No datasets were generated or analysed during the current study.
